# Psychometric evaluation of the problematic internet use questionnaire in Chinese emerging adults: an exploratory alternative three-factor structure and unidimensional short forms

**DOI:** 10.3389/fpubh.2026.1980618

**Published:** 2026-10-26

**Authors:** Sai-Fu Fung, Cyrus Ngo Tin Lai, Jessica Garneau, Annick Parent-Lamarche

**Affiliations:** 1 Department of Social and Behavioural Sciences, City University of Hong Kong, Hong Kong, Hong Kong SAR, China; 2 Jockey Club School of Public Health and Primary Care, The Chinese University of Hong Kong, Hong Kong, New Territories, Hong Kong SAR, China; 3 Human Resources Management Department, Business School, Université du Québec à Trois-Rivières, Trois-Rivières, QC, Canada

**Keywords:** CFA, EFA, internet addiction, problematic internet and technology use, problematic internet entertainment use, psychometric, university student

## Abstract

**Introduction:**

Problematic internet use (PIU) is a growing concern among university students. The 18-item Problematic Internet Use Questionnaire (PIUQ-18) is widely used, yet its factor structure remains debated across cultures. This study examined the psychometric properties of the PIUQ-18 and its short forms, the PIUQ-SF-9 and PIUQ-SF-6, among Chinese emerging adults.

**Methods:**

Participants were 511 Chinese emerging adults (85.50% female). A split-sample design was used, with *n* = 260 *n* = 260 for exploratory factor analysis (EFA) and *n* = 251 *n* = 251 for confirmatory factor analysis (CFA). We evaluated internal consistency, factor structure, and construct validity, including associations with well-being indicators.

**Results:**

The PIUQ-18 demonstrated good internal consistency (α = 0.87 α = 0.87, ω = 0.87 ω = 0.87). The original three-factor model produced latent correlations exceeding 1.00 (Obsession–Control = 1.05; Neglect–Control = 1.09), rendering it statistically inadmissible in this sample. An alternative EFA-derived three-factor model was the best-fitting admissible PIUQ-18 model (CFI = 0.91, TLI = 0.90, RMSEA = 0.07, SRMR = 0.07), although its incremental fit remained below conventional benchmarks. HTMT values were below conventional thresholds, but confidence intervals for the latent correlations indicated that the dimensions remained substantially related. For the short forms, the PIUQ-SF-6 demonstrated acceptable unidimensional fit (CFI = 0.97, TLI = 0.95, RMSEA = 0.08, SRMR = 0.05), and the PIUQ-SF-9 showed comparable but weaker fit (CFI = 0.91, TLI = 0.88, RMSEA = 0.11, SRMR = 0.07). The Control Disorder subscale exhibited weaker reliability and validity and requires refinement.

**Discussion:**

The original three-factor structure of the PIUQ-18 was not statistically admissible in this sample. An alternative EFA-derived three-factor structure showed promise, but its incremental fit was below conventional benchmarks, and the dimensions remained substantially related. The Control Disorder subscale showed weaker reliability and validity, indicating a need for refinement. The PIUQ-SF-6 showed preliminary support for a unidimensional factor structure and theoretically consistent associations with well-being indicators, whereas the PIUQ-SF-9 showed comparable but weaker fit. Overall, these findings should be interpreted as preliminary evidence rather than as evidence establishing screening utility.

## 1 Introduction

With rapid technological advancements, the Internet has evolved into an essential part of daily life. The Internet offers people new ways of communicating, educating, and entertaining (1). However, the increasing integration of the Internet into daily life has also contributed to the emergence of problematic internet use (PIU) as a growing public health concern. PIU is generally defined as excessive or poorly controlled internet use that leads to significant psychological, social, academic, occupational, or physical impairment (2). Emerging adults, particularly university students, are at high risk of developing PIU (3). This developmental period is characterized by reduced parental supervision and greater access to internet resources on university campuses (4, 5). University students face unique transitional stressors, including intense academic pressures and identity formation challenges, which may increase vulnerability to using the internet as a coping mechanism and contribute to PIU patterns (6).

The Problematic Internet Use Questionnaire (PIUQ) is a widely used instrument for identifying PIU. Developed based on addiction theory, it assesses three core dimensions: Obsession (obsessive thinking about the internet and withdrawal symptoms), Neglect (neglecting daily activities and social relationships), and Control Disorder (inability to control internet use) (7). The original 18-item version (PIUQ-18), along with its shorter forms (PIUQ-SF-9 and PIUQ-SF-6), has demonstrated strong psychometric properties and has been validated in diverse cultural contexts, including Hungary, the United States, China, Japan, several European countries (e.g., Italian, German, French, Polish, Turkish, Greek), Portugal, Nigeria, and Brazil (7–16). However, validation studies specifically in Chinese populations remain relatively limited, highlighting the need for further research in this context.

Apart from the limited studies, debates continue regarding PIUQ’s optimal factor structure in different versions. The original PIUQ-18 consists of 18 items across three theoretically derived factors: Obsession, Neglect, and Control Disorder, with six items per factor, and this three-factor model has received empirical support in prior work (7, 9). The PIUQ-SF-9 is a nine-item short form that retains the same three-factor framework but uses three items for each dimension, whereas the PIUQ-SF-6 is a six-item brief form that also reflects the same conceptual structure but includes only two items per factor. However, several PIUQ-SF-9 studies have suggested that these three dimensions are highly correlated, potentially reflecting a dominant general PIU factor rather than clearly separable subdimensions (8, 13, 16). These studies have therefore examined both correlated factor models and bifactor models that specify a general factor alongside specific dimension. In contrast, the only Chinese validation study of the PIUQ-SF-9 indicated that the three-factor model fit best, leaving room to revisit its structure in Chinese samples (12). For the PIUQ-SF-6, evidence is even more limited, although most studies have favored a three-factor solution over a one-factor model (9, 15). To the best of our knowledge, no Chinese validation study has examined the PIUQ-SF-6. These findings motivate us to explore the factor structure of different PIUQ versions in a sample of Chinese emerging adults.

The present study evaluated the PIUQ-18 in Chinese university students using a split-sample design and examined the PIUQ-SF-9 and PIUQ-SF-6 derived from the same item pool. The primary objective was to investigate whether the previously proposed PIUQ structures were statistically admissible and substantively interpretable in this sample. A secondary objective was to examine whether the short forms showed evidence consistent with unidimensional scoring. In evaluating construct validity, the present study distinguishes between convergent associations with related indicators and discriminant validity among PIUQ dimensions. High correlations among latent factors may indicate that the dimensions are strongly related, but correlations below 1.0 alone do not establish that the constructs are empirically distinct. Accordingly, discriminant validity should be evaluated using additional criteria, such as the heterotrait–monotrait ratio and confidence intervals for factor correlations, when the available data and model estimation permit.

## 2 Methods

### 2.1 Participants

This study involved 511 university students with a mean age of 20.41 years (*SD* = 2.49) in China, representing the emerging adult population enrolled in university. Data was collected between April and May 2019 through the university’s student intranet platform. Recruitment was voluntary, and no random, stratified, or probability-based sampling procedure was used. The resulting sample should therefore be characterized as a single-university voluntary convenience sample rather than a representative university sample. All responses were collected anonymously to safeguard participants’ privacy, and no identifying information was retained. Participation was entirely voluntary; informed consent was obtained before data collection, and individuals could withdraw at any time. This study was conducted in accordance with the Declaration of Helsinki and approved by the Institutional Review Board of a university in Hong Kong.

Eligible participants were university students who could understand Chinese and provided informed consent. Those unable to comprehend Chinese or who refused consent were excluded. The data were collected specifically for this psychometric validation and did not originate from any existing institutional records. The 511 respondents represented approximately 2.56% of the estimated university population of 20,000 students. Because the intranet distribution system did not provide a verified denominator of eligible students who received, opened, or were exposed to the invitation, this percentage should not be interpreted as a conventional individual-level survey response rate. The low participation proportion and voluntary recruitment may have introduced self-selection and nonresponse bias.

Sample-size planning considered both conventional participant-to-item guidelines and the complexity of the planned factor analyses. No formal *a priori* statistical power analysis was conducted. For the exploratory factor analysis, a commonly cited rule of thumb recommends approximately 10 participants per item. For the 18-item PIUQ-18, this corresponds to a target of approximately 180 participants. The EFA subsample included 260 participants (Sample 1), corresponding to a participant-to-item ratio of 14.4:1 and therefore exceeding this conventional 10:1 guideline. However, participant-to-item ratios are heuristic rather than universal requirements. Simulation studies indicate that the adequacy of an EFA sample depends additionally on communalities, factor loadings, the number of indicators per factor, and the degree to which each factor is overdetermined (17, 18). In the present EFA, the 18 items were examined across a three-factor solution, with multiple indicators defining each factor and several moderate-to-strong loadings. These features provided additional support for the planned EFA sample, although the exploratory solution still requires replication in an independent sample.

The CFA subsample comprised 251 participants, corresponding to approximately 14 participants per item for the 18-item PIUQ-18. This exceeded the conventional 10:1 participant-to-item planning heuristic and the preliminary N = 200 target used during study planning. Nevertheless, participant-to-item ratios do not establish statistical power or guarantee stable estimation for all CFA models. Simulation studies indicate that sample-size requirements depend on model complexity, loading magnitudes, communalities, factor overdetermination, estimator, and the risk of improper solutions (17–19). The present sample was therefore considered reasonable for the relatively simple one-factor and correlated-factor models, but potentially less adequate for bifactor models and subgroup measurement-invariance analyses.

The split-sample allocation was determined before the primary analyses to reduce capitalizing on chance during confirmatory testing. Nevertheless, the EFA and CFA subsamples were drawn from the same recruitment pool and are therefore not independent samples in the population-replication sense. The CFA provides internal cross-validation within this dataset, but independent-sample replication remains necessary.

### 2.2 Measure

The Problematic Internet Use Questionnaire (PIUQ-18) is an 18-item self-report instrument designed to assess PIU. In the present study, the PIUQ-18 was translated into Simplified Chinese following established cross-cultural adaptation guidelines (20, 21). Two bilingual translators with postgraduate degrees in translation independently produced forward translations from English to Chinese. These two versions were synthesized by a third bilingual expert to produce a reconciled Chinese version. Two different bilingual translators, blinded to the original English version, then independently back-translated the reconciled Chinese version into English. An expert committee reviewed all versions and assessed semantic, idiomatic, experiential, and conceptual equivalence. Although a Simplified Chinese PIUQ-SF-9 exists (12), we retranslated the full PIUQ-18 to ensure consistency across the full scale and both short forms. A pilot study was conducted with 15 participants from the target population (university students) who completed the questionnaire. Responses were made on a 5-point Likert scale (1 = never to 5 = always), with higher total scores indicating greater PIU severity. Supplementary analyses were also conducted to examine the two shorter versions of PIUQ-18, which are PIUQ-SF-9 and PIUQ-SF-6, consistent with prior literature showing that short forms can be meaningfully evaluated from the full item pool when their structural performance is explicitly tested (8, 9, 12). No items were identified as difficult to understand. Data from the pilot study was not included in the primary analysis.

The 6-item Brief Resilience Scale (BRS) (22) was used to measure resilience, specifically the ability to recover from stress and bounce back from adversity. Sample items include: “I tend to bounce back quickly after hard times” and “It is hard for me to snap back when something bad happens” (reverse-scored). Responses are rated on a 5-point Likert scale (1 = strongly disagree to 5 = strongly agree). The BRS demonstrates good to excellent internal consistency (α = 0.80–0.91) across various populations (22). The Chinese version was recently validated by Fung (23).

The 10-item Rosenberg Self-Esteem Scale (RSE) (24) was applied to evaluate global self-esteem. Sample items include: “On the whole, I am satisfied with myself” and “At times I think I am no good at all” (reverse scored). Participants rate each item on a 4-point scale (1 = strongly disagree to 4 = strongly agree). The RSE is one of the most widely validated measures of self-esteem and has shown consistent internal reliability (α = 0.75–0.88) in diverse samples (25). Leung and Wong (26) validated the Chinese version.

The 4-item Subjective Happiness Scale (SHS) (27) was used to measure participants’ global subjective happiness. It assesses happiness through self-evaluations and comparisons with others. Sample items include: “In general, I consider myself” (rated from 1 = not a very happy person to 7 = a very happy person) and “Some people are generally very happy. They enjoy life regardless of what is going on, getting the most out of everything. To what extent does this characterization describe you?” The SHS demonstrates good to excellent internal consistency (α = 0.86) (28). The Chinese version, validated previously (29), was used in this study.

### 2.3 Data analysis

Analyses were conducted using R (Version 4.3.1). To strengthen the robustness of the analyses and avoid overfitting, the sample was randomly divided into two groups. Sample 1 (*n* = 260) was used for descriptive and exploratory factor analyses, whereas Sample 2 (*n* = 251) was designated for CFA and construct validity. Employing separate samples for EFA and CFA aligns with methodological standards recommended by Fokkema and Greiff (30).

Internal consistency was evaluated using Cronbach’s alpha, corrected item-total correlations, alpha-if-item-deleted values, and McDonald’s omega (31). Construct validity among the PIUQs, the Rosenberg Self-Esteem Scale (RSE), Subjective Happiness Scale (SHS), and Brief Resilience Scale (BRS) were examined using Pearson correlations.

EFA was conducted using principal axis factoring (PAF) with polychoric correlations and oblique (Oblimin) rotation. This approach was selected for three reasons: (1) PAF does not assume multivariate normality and is widely recognized as appropriate for ordinal Likert-scale data (32); (2) polychoric correlations are specifically designed for ordinal data and align with the WLSMV estimator used in subsequent CFA (33); and (3) the constructs are theoretically expected to correlate. As a sensitivity analysis, we also examined a varimax-rotated solution to facilitate comparison with previous PIUQ studies (9, 10, 14, 15). The overall factor pattern was similar across rotations. Factorability was assessed using the Kaiser-Meyer-Olkin measure and Bartlett’s test of sphericity.

CFA was estimated to use the weighted least squares mean- and variance-adjusted (WLSMV) estimator because the items were treated as ordered categorical variables, an approach well supported for ordinal psychometric data (33). We tested five competing models: (1) a one-factor model, (2) the original three-factor model [Demetrovics’ three-factor model; (10)], (3) a new three-factor model derived from our EFA results, and bifactor models with two specific and three specific factors (8, 13, 16). Model fit was evaluated using chi-square, comparative fit index (CFI), Tucker-Lewis index (TLI), root mean square error of approximation (RMSEA), and standardized root mean square residual (SRMR). Model fit was evaluated using the chi-square statistic, CFI, TLI, RMSEA with its 90% confidence interval, and SRMR. These indices were interpreted jointly rather than according to rigid universal cutoffs. Approximate benchmarks were considered in conjunction with parameter admissibility, loading magnitudes, factor correlations, residuals, model complexity, and substantive interpretability. Because the data were ordinal and the models were estimated using WLSMV, fit indices were interpreted cautiously and were not treated as sufficient evidence of substantive validity. Non-positive-definite covariance matrices, negative residual variances, and nonconvergence were treated as improper solutions that could not be interpreted reliably. Measurement invariance across gender was not tested. Although gender invariance would be relevant to determining whether PIUQ scores can be compared across groups, the sample was highly imbalanced, with 85.5% female participants. The available subgroup sizes were not considered sufficient for stable estimation and interpretation of ordinal configural, loading, threshold, and scalar invariance models. Gender invariance was therefore reserved for future research using larger and more balanced samples.

Discriminant validity of the multidimensional models (both the original Demetrovics three-factor model and the EFA-derived alternative three-factor model) was examined using two complementary approaches. First, 95% confidence intervals for all latent factor correlations were computed using the delta method, based on the standardized solution output of the fitted CFA models. The delta method approximates the standard error of each standardized correlation from the model-implied covariance matrix and the asymptotic covariance matrix of the parameter estimates, and the 95% CI is obtained as the standardized estimate ± 1.96 × delta-method standard error. This approach is appropriate here because the WLSMV estimator provides a consistent asymptotic covariance matrix for ordered categorical data, and because bootstrap resampling was not used. A model was considered admissible only if every latent correlation fell strictly below 1.00; correlations at or above this bound indicate a non-positive-definite latent covariance matrix and render the solution statistically inadmissible (34). Second, the heterotrait–monotrait ratio of correlations (HTMT) was computed from the polychoric correlation matrix following the procedure described by Henseler et al. (35). For each pair of factors, HTMT was defined as the mean correlation between items measuring different factors (heterotrait) divided by the geometric mean of the average correlations among items measuring the same factor (monotrait). HTMT values below 0.85 were taken as evidence of discriminant validity; values at or above 0.85 (or 0.90 for conceptually similar constructs) indicate discriminant-validity problems, and values at or above 1.00 are mathematically incompatible with a proper solution.

### 2.4 Transparency and openness

This study’s design and analysis were not preregistered. The de-identified data, analytic code (R syntax), and research materials are not publicly available but can be obtained upon reasonable request from the corresponding author. Data were analyzed using R, Version 4.3.1, and the following packages: *lavaan*, Version 0.6–0.15 (36); and *psych*, Version 2.3.3 (37).

### 2.5 Ethical considerations

The authors declare that the entire research procedure was conducted in accordance with the ethical standards of City University of Hong Kong research ethics committee (approval number: SS6591) and with the 1964 Helsinki declaration and its later amendments. Informed written consent was obtained from the study participants.

## 3 Results

### 3.1 Descriptive statistics and internal consistency

Internal consistency reliability of the original PIUQ-18 was examined in Sample 1 (*n* = 260). The PIUQ-18 demonstrated good internal consistency and reliability. Cronbach’s alpha was 0.87 (95% CI [0.85, 0.89]); McDonald’s omega was 0.87, reflecting satisfactory internal consistency (38). The average inter-item correlation was 0.27. As shown in Table 1, corrected item-total correlations (*r*_it_) ranged from 0.28 (PIUQ1) to 0.62 (PIUQ13), with most items showing acceptable values (> 0.40). Alpha-if-item-deleted (αiid) statistics indicated that removing any single item did not substantially improve reliability; the highest alpha after deletion remained 0.87 (after removing PIUQ1). Item descriptive statistics revealed acceptable variability (*SD*s ranging from 0.84 to 1.12), and no items exhibited extreme skewness or floor/ceiling effects. Item means ranged from 2.1 (PIUQ2 and PIUQ11) to 3.3 (PIUQ1 and PIUQ9). Skewness values ranged from −0.82 to 0.95, and kurtosis from −0.93 to 0.55, indicating distributions within acceptable limits for factor analysis (39).

**Table 1:** Descriptive statistics, item properties, and factor loadings from the principal axis factoring EFA with oblique oblimin rotation.

Item	*M*	*SD*	Skew	Kurt	*r*_it_	$\alpha_{\mathrm{iid}}$	Preocc	Neg	Con	h^2^	u^2^
PIUQ1	3.30	0.84	−0.82	0.45	0.28	0.87	—	—	0.62	0.29	0.71
PIUQ2	2.10	1.02	0.68	−0.41	0.41	0.87	—	0.33	—	0.24	0.76
PIUQ3	2.20	0.86	0.35	−0.52	0.32	0.87	—	0.68	—	0.40	0.60
PIUQ4	3.10	0.90	−0.41	−0.38	0.45	0.87	—	—	0.54	0.36	0.64
PIUQ5	2.70	0.97	0.12	−0.71	0.60	0.86	0.53	—	—	0.49	0.51
PIUQ6	2.50	0.99	0.28	−0.65	0.57	0.86	0.63	—	—	0.48	0.52
PIUQ7	2.90	0.94	−0.09	−0.68	0.41	0.87	0.57	—	—	0.32	0.68
PIUQ8	1.90	0.98	0.95	0.55	0.56	0.86	0.71	—	—	0.51	0.49
PIUQ9	3.30	0.87	−0.51	−0.12	0.40	0.87	0.55	—	—	0.27	0.73
PIUQ10	2.40	1.01	0.41	−0.59	0.57	0.86	0.32	0.38	—	0.45	0.55
PIUQ11	2.10	0.92	0.71	−0.08	0.48	0.87	—	0.59	—	0.45	0.55
PIUQ12	3.10	0.92	−0.47	−0.21	0.59	0.86	—	—	0.68	0.58	0.42
PIUQ13	2.60	1.12	0.19	−0.93	0.62	0.86	0.35	0.33	—	0.49	0.51
PIUQ14	3.00	1.00	−0.12	−0.71	0.34	0.87	—	—	0.47	0.23	0.77
PIUQ15	2.50	1.07	0.22	−0.84	0.34	0.86	0.60	—	—	0.42	0.58
PIUQ16	3.10	0.92	−0.47	−0.21	0.57	0.86	—	—	0.64	0.58	0.42
PIUQ17	2.40	1.01	0.38	−0.55	0.54	0.86	—	0.62	—	0.50	0.50
PIUQ18	2.60	1.11	0.09	−0.88	0.52	0.86	—	0.41	—	0.39	0.61

Skew, Skewness; Kurt, Kurtosis; r_it_, Corrected item-total correlations; αiid, Cronbach’s alpha, if item deleted; h^2^, communality; u^2^, uniqueness (unique/specific variance); Preocc, Preoccupation; Neg, Neglect; Con, Control Disorder.

### 3.2 Exploratory factor analysis across PIUQ versions

EFA was conducted on Sample 1 (*n* = 260) using principal axis factoring (PAF) with polychoric correlations and oblimin (oblique) rotation. Factorability was excellent across scales, as shown in Table 2: PIUQ-18 (KMO = 0.88, Bartlett’s χ^2^(153) = 1388.8, *p* < 0.001), PIUQ-SF-9 (KMO = 0.84, Bartlett’s χ^2^(36) = 446.7, *p* < 0.001), and PIUQ-SF-6 (KMO = 0.80, Bartlett’s χ^2^(15) = 301.0, *p* < 0.001).

**Table 2:** Exploratory factor analysis results across PIUQ versions.

Scale	Model	BIC	RMSR	Variance explained
PIUQ-18	1-factor	−220	0.08	31.9%
PIUQ-18	2-factor	−261	0.06	32.7%
PIUQ-18	3-factor	−245	0.05	35.2%
PIUQ-SF-9	1-factor	−83	0.06	31.9%
PIUQ-SF-6	1-factor	−30	0.05	37.6%

BIC, Bayesian Information Criterion; RMSR, Root Mean Square Residual.

Parallel analysis suggested that a four-factor solution warranted consideration, whereas the scree plot showed a clear elbow at three factors (see Figure 1). The observed eigenvalues decreased from Factor 1 (6.38) to Factor 2 (1.61) and Factor 3 (1.22), followed by a relatively flat pattern beginning with Factor 4 (1.10). However, the four-factor solution was not retained for two reasons. First, the additional factor was defined primarily by a single salient indicator (PIUQ8, loading = 0.95), making it difficult to interpret as a substantive dimension. Second, the solution produced a Heywood case, with PIUQ8 showing a communality of 1.07, exceeding the theoretical maximum of 1.00, which indicates empirical over-extraction and an improper solution. By contrast, the three-factor solution showed no communality exceeding 1.00 (maximum = 0.58). The three-factor solution therefore provided the most interpretable and admissible configuration.

**Figure 1:**
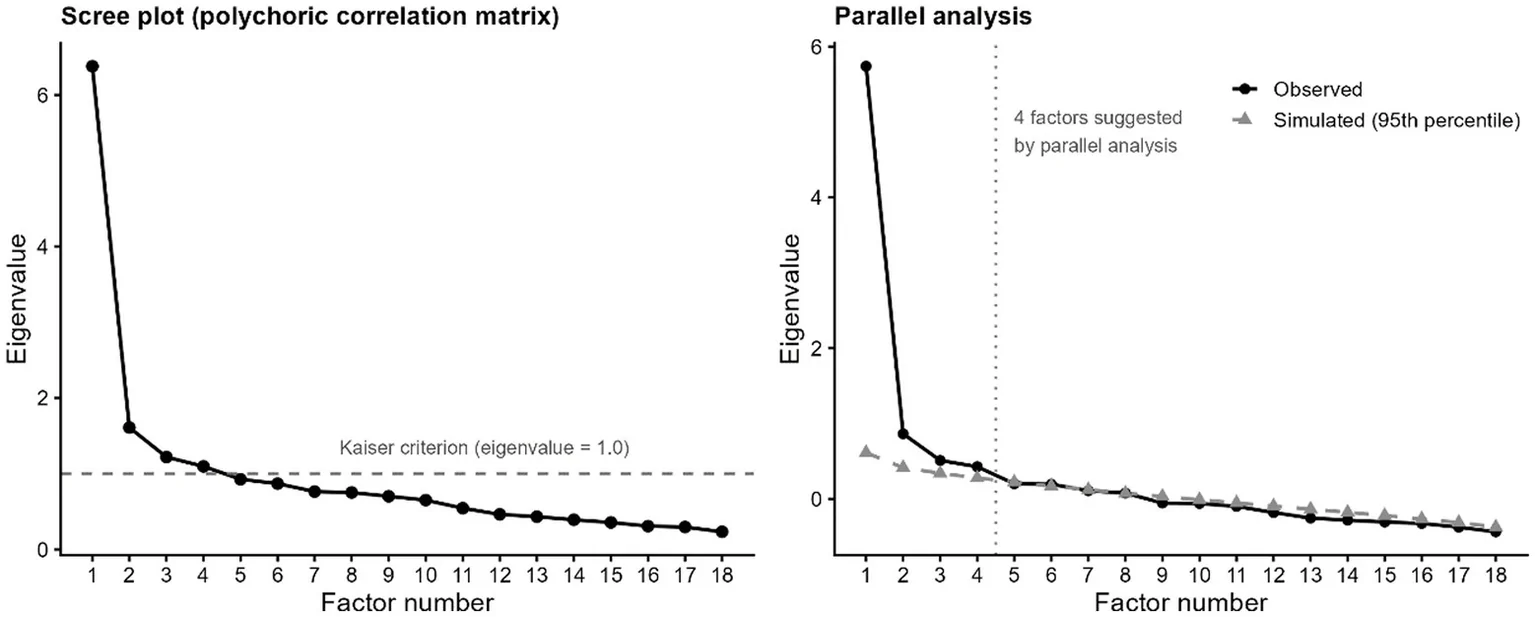
Scree plot and parallel analysis for PIUQ-18.

The three-factor solution with oblimin rotation was interpretable, although PIUQ10 and PIUQ13 showed cross-loadings that warrant caution in interpreting the factor assignments. It explained 35.2% of the total variance. The three factors were labeled Preoccupation, Neglect, and Control based on their dominant item content. Item assignments and loadings are presented in Table 1. Factor correlations were Φ = 0.61 (Preoccupation–Neglect), 0.56 (Preoccupation–Control), and 0.36 (Neglect–Control), confirming that the dimensions are correlated and supporting the use of an oblique rotation. Cross-loadings were observed for Items 10 and 13. Because PIUQ13 loaded only marginally higher on Preoccupation than on Neglect, it should be regarded as unstable and is a candidate for revision in future work.

For the PIUQ-SF-9, parallel analysis suggested retaining one factor. The one-factor solution explained 31.90% of the total variance. All items loaded at or above 0.30, with the strongest loadings observed for item 13 (0.75), item 5 (0.73), and item 6 (0.69). Communalities ranged from 0.13 to 0.57. For the PIUQ-SF-6, parallel analysis also supported retaining one factor. The one-factor solution showed the strongest loadings for item 5 (0.76), item 6 (0.75), and item 13 (0.73), with communalities ranging from 0.12 to 0.57. The higher proportion of variance explained by the SF-6, relative to the SF-9 and to the PIUQ-18 three-factor solution, reflects the smaller item pool rather than a stronger general factor per se, and should be interpreted alongside the confirmatory fit indices reported in Table 3. Overall, the EFA suggested that a multidimensional structure was plausible for the full scale (PIUQ-18), while the short forms (PIUQ-SF-9 and PIUQ-SF-6) converged on a stable unidimensional solution. However, confirmatory testing (CFA) is required to determine whether the multidimensional structure is statistically admissible.

**Table 3:** Confirmatory factor analysis results across PIUQ versions.

Item	PIUQ-18							PIUQ-SF-9	PIUQ-SF-6
	1-Factor	Demetrovics’ three-factor			EFA-derived three-factor			1-Factor	1-Factor
		Obs	Neg	Con	Preocc	Neg	Con		
PIUQ1	0.37	0.38	–	–	–	–	0.46	–	–
PIUQ2	0.56	–	0.56	–	–	0.59	–	0.5	–
PIUQ3	0.56	–	–	0.54	–	0.58	–	0.5	–
PIUQ4	0.49	0.50	–	–	–	–	0.58	–	–
PIUQ5	0.58	–	0.57	–	0.66	–	–	0.69	0.71
PIUQ6	0.66	–	–	0.64	0.76	–	–	0.77	0.79
PIUQ7	0.38	0.38	–	–	0.45	–	–	0.49	0.55
PIUQ8	0.59	–	0.58	–	0.67	–	–	0.35	0.38
PIUQ9	0.37	0.56	–	0.35	0.42	–	–	0.35	0.38
PIUQ10	0.56	–	0.56	–	–	0.57	–	0.53	–
PIUQ11	0.60	–	–	0.60	0.63	–	–	–	–
PIUQ12	0.66	0.63	–	0.63	–	0.79	0.63	0.65	–
PIUQ13	0.61	0.62	–	–	–	0.63	–	0.63	0.65
PIUQ14	0.42	–	0.42	–	–	–	0.50	0.28	0.24
PIUQ15	0.45	0.42	–	0.43	0.49	–	–	–	–
PIUQ16	0.55	0.56	–	–	–	–	0.65	–	–
PIUQ17	0.60	–	0.59	–	–	0.62	–	–	–
PIUQ18	0.57	–	–	0.55	–	0.59	–	–	–
RMSEA	0.09	0.09			0.07			0.11	0.08
RMSEA (90% CI)	[0.08, 0.10]	[0.08, 0.10]			[0.06, 0.08]			[0.08, 0.13]	[0.04, 0.12]
SRMR	0.08	0.08			0.07			0.07	0.05
χ^2^	430.1	422.3			306			100.8	22.8
*df*	135	132			132			27	9
χ^2^/*df*	3.19	3.2			2.32			3.73	2.54
CFI	0.85	0.86			0.91			0.91	0.97
TLI	0.83	0.83			0.9			0.88	0.95

Obs, Obsession; Neg, Neglect; Con, Control Disorder; Preocc, Preoccupation. All loadings were significant at *p* < 0.001.

### 3.3 Confirmatory factor analysis across PIUQ versions and discriminant validity

CFA was conducted on Sample 2 (*n* = 251), shown in Table 3. The prespecified one-factor, original three-factor, EFA-derived alternative three-factor models, and bifactor models with three specific factors and two specific factors were examined.

The one-factor PIUQ-18 model showed poor fit, χ^2^(135) = 430.11, χ^2^/df = 3.19, CFI = 0.85, TLI = 0.83, RMSEA = 0.09 [0.08, 0.10], and SRMR = 0.08. The Demetrovics’ three-factor model showed a similar fit to the one-factor model, with CFI = 0.86, TLI = 0.83, RMSEA = 0.09 [0.08, 0.10], and SRMR = 0.08. However, two latent correlations exceeded 1.0: Obsession–Control (*r* = 1.05, 95% CI [0.98, 1.12]) and Neglect–Control (*r* = 1.09, 95% CI [1.02, 1.16]). The correlation between Obsession and Neglect was also very high (*r* = 0.95, 95% CI [0.87, 1.03]). These estimates indicate a non-positive-definite latent covariance matrix, rendering the model statistically inadmissible, and the fit indices are therefore not interpretable as evidence of fit. Discriminant validity for this model was assessed using the heterotrait–monotrait ratio of correlations (HTMT; Table 4). All three HTMT values exceeded the 0.90 threshold: Obsession–Neglect = 0.97, Obsession–Control = 1.05, and Neglect–Control = 1.05. Two values exceeded 1.00, which is mathematically incompatible with a proper solution and corroborates the inadmissibility reported above. The original Demetrovics three-factor model was therefore rejected.

**Table 4:** Discriminant-validity diagnostics for the PIUQ-18 three-factor models.

Model/factor pair	*r*	95% CI	HTMT
Original Demetrovics 3-factor			
Obsession—Neglect	0.95	[0.87, 1.03]	0.97
Obsession—Control	1.05	[0.98, 1.12]	1.05
Neglect—Control	1.09	[1.02, 1.16]	1.05
EFA-derived 3-factor			
Preoccupation—Neglect	0.80	[0.73, 0.87]	0.79
Preoccupation—Control	0.53	[0.42, 0.65]	0.52
Neglect—Control	0.76	[0.68, 0.83]	0.75

HTMT, heterotrait–monotrait ratio of correlations, computed from the polychoric correlation matrix.

In contrast, the EFA-derived three-factor model demonstrated comparatively better fit, with CFI = 0.91, TLI = 0.90, RMSEA = 0.07 [0.06, 0.08], and SRMR = 0.07. The post-estimation check passed, and all latent correlations were below 1.00: Preoccupation–Neglect (*r* = 0.80, 95% CI [0.73, 0.87]), Preoccupation–Control (*r* = 0.53, 95% CI [0.42, 0.65]), and Neglect–Control (*r* = 0.76, 95% CI [0.68, 0.83]). All three corresponding HTMT values were below the 0.85 threshold (Preoccupation–Neglect = 0.79; Preoccupation–Control = 0.52; Neglect–Control = 0.75; Table 4), providing preliminary evidence consistent with discriminant validity. This model was statistically admissible and showed the most favorable fit among the admissible models tested, although its incremental fit indices remained below conventional thresholds (CFI/TLI ≥ 0.95), and it should therefore be regarded as provisional rather than definitive.

For the PIUQ-SF-9, the one-factor model showed acceptable fit, with CFI = 0.91, TLI = 0.88, RMSEA = 0.11 [0.08, 0.13], and SRMR = 0.07. The three-factor model showed a slightly better chi-square but poorer RMSEA (0.11 [0.09, 0.13]) and produced an improper solution. Therefore, the one-factor model was retained as the best-supported admissible solution for the PIUQ-SF-9. In this model, standardized factor loadings ranged from 0.28 (PIUQ14) to 0.77 (PIUQ6), with PIUQ14 showing a notably low loading.

Finally, the one-factor model of PIUQ-SF-6 demonstrated acceptable fit (CFI = 0.97, TLI = 0.95, RMSEA = 0.08 [0.04, 0.12], and SRMR = 0.05), establishing it as the optimal solution consistent with EFA findings. Although a three-factor model showed marginally better SRMR (0.05) and chi-square, it produced a non-positive definite covariance matrix, rendering parameter estimates unreliable. Bifactor models (three-specific and two-specific) similarly failed to converge or yielded improper solutions with negative variances, confirming empirical non-identification. Standardized loadings in the one-factor PIUQ-SF-6 ranged from 0.24 (PIUQ14) to 0.79 (PIUQ6). Unlike the PIUQ-18’s inadmissible multi-factor structure, PIUQ-SF-6 proved the most stable and parsimonious measure, with PIUQ-SF-9 as a reasonable but longer alternative.

### 3.4 Construct validity

Construct validity of the PIUQs was examined in Sample 2 (*n* = 251) by computing Pearson correlation coefficients between the total scores of the PIUQ-18, PIUQ-SF-6, and PIUQ-SF-9 with various well-being indicators (Table 5). The PIUQ-SF-9 demonstrated excellent convergence with the full PIUQ-18 (*r* = 0.92, *p* < 0.001) and with the PIUQ-SF-6 (*r* = 0.92, *p* < 0.001). The PIUQ-SF-6 also showed strong convergence with the PIUQ-18 (*r* = 0.83, *p* < 0.001). When examining non-overlapping correlations (correlating each short form with the remaining PIUQ-18 items not included in that short form), the PIUQ-SF-9 showed *r* = 0.70 and the PIUQ-SF-6 showed *r* = 0.60, providing more conservative estimates of shared variance.

**Table 5:** Pearson correlations across PIUQs, BRS, RSE, and SHS.

	PIUQ-18				PIUQ-SF-9	PIUQ-SF-6
	1-Factor	EFA-derived three-factor			1-Factor	1-Factor
Variables	General	Preocc	Neg	Con	General	General
BRS	−0.28***	−0.24**	−0.30***	−0.12	−0.23***	−0.18**
RSE	−0.21**	−0.17	−0.19*	−0.14	−0.16**	−0.15*
SHS	−0.22***	−0.17	−0.22**	−0.13	−0.19**	−0.14*

*p < 0.05, **p < 0.01, ***p < 0.001.

The short forms exhibited theoretically coherent associations with external criteria. They showed consistent negative associations with multiple well-being measures, including resilience (BRS: *r* = −0.28, *p* < 0.001 for PIUQ-18; *r* = −0.18, *p* < 0.01 for PIUQ-SF-6; and r = −0.23, *p* < 0.001 for PIUQ-SF-9), self-esteem (RSE: *r* = −0.21, *p* < 0.01 for PIUQ-18; *r* = −0.15, *p* < 0.05 for PIUQ-SF-6; and r = −0.16, *p* < 0.01 for PIUQ-SF-9), and subjective happiness (SHS: *r* = −0.22, *p* < 0.001 for PIUQ-18; *r* = −0.14, *p* < 0.05 for PIUQ-SF-6; and *r* = −0.19, *p* < 0.01 for PIUQ-SF-9). These patterns were highly similar in magnitude and direction to those observed for the full PIUQ-18, supporting the construct validity of the short forms.

To further examine the construct validity of the newly identified three-factor model, we computed correlations between its three subscales (Preoccupation, Neglect, Control Disorder) and the well-being indicators. The Preoccupation and Neglect subscales showed theoretically consistent patterns, with significant negative associations with resilience (*r* = −0.24 and −0.30, respectively), self-esteem (*r* = −0.17 and −0.19), and subjective happiness (*r* = −0.17 and −0.22). In contrast, the Control Disorder subscale showed weaker and mostly non-significant associations with all well-being measures (*r*s ranging from −0.14 to −0.12). The correlations among the three subscales ranged from 0.37 to 0.58, indicating moderate overlap.

## 4 Discussion

The findings provide mixed support for PIUQ’s proposed multidimensional structure. The PIUQ-18 showed satisfactory internal consistency. The EFA supported a three-factor pattern, but the original Demetrovics item configuration was not admissible in CFA because two latent correlations exceeded 1.0. An alternative EFA-derived configuration showed better fit and acceptable latent correlations. However, this new three-factor model’s alternative structure does not align with previously published PIUQ factor structures, and its emergence in this sample may reflect cultural or contextual factors in how PIU symptoms are experienced by Chinese emerging adults. While previous cross-cultural validation studies have reported alternative factor structures for the PIUQ (7, 10, 11, 29), none have identified this specific item assignment pattern. Therefore, the EFA-derived three-factor model should be considered an exploratory structure requiring cross-validation in independent Chinese samples before it can be recommended for widespread use. Although this alternative was statistically admissible and showed better fit than the original Demetrovics structure, statistical admissibility is not equivalent to substantive validity. The alternative structure emerged from this sample, does not match any previously published PIUQ configuration, and its Control Disorder dimension showed weak reliability and weak external associations. It should therefore be regarded as a provisional exploratory model rather than a validated alternative.

At the subscale level, construct validity analyses revealed important nuances in the EFA-derived three-factor model. The Preoccupation and Neglect subscales demonstrated theoretically coherent associations with well-being measures, showing significant negative correlations with resilience (*r* = −0.24 and −0.30, respectively), self-esteem (*r* = −0.17 and −0.19), and subjective happiness (*r* = −0.17 and −0.22). These patterns support the construct validity of these two dimensions, as higher levels of preoccupation and neglect were associated with poorer psychological well-being. In contrast, the Control Disorder subscale showed weaker and mostly non-significant associations with all well-being measures (ranging from −0.14 to −0.12). Additionally, the internal consistency of the Control Disorder subscale was somewhat lower (α = 0.70) compared to Preoccupation (α = 0.71) and Neglect (α = 0.75). The correlations among the three subscales ranged from 0.37 to 0.58, indicating moderate overlap but sufficient distinctiveness to justify retaining the three-factor structure. These findings provide preliminary support for the interpretability of the Preoccupation and Neglect dimensions, whereas the Control Disorder dimension requires further refinement and independent validation.

In contrast, the short forms behaved consistently with the full scale. Both PIUQ-SF-9 and PIUQ-SF-6 were best represented by a one-factor model in the confirmatory analyses, and the one-factor model was the only admissible solution for both short forms. This finding is consistent with the EFA results on Sample 1, where parallel analysis supported a single general PIU factor for both short versions. The three-factor models for the short forms, while tested for theoretical consistency, produced improper solutions (non-positive definite latent covariance matrices) due to extremely high inter-factor correlations. These results suggest that, with fewer items, the PIUQ-SF-6 and PIUQ-SF-9 primarily capture a dominant general PIU factor in this Chinese university sample, even though they were originally derived from the three-dimensional framework. This pattern for the short forms converges with several studies that have reported strong general-factor dominance in bifactor solutions for the PIUQ-SF-9 (8, 13, 16). These findings differ from the earlier Chinese validation of the PIUQ-SF-9, which supported the three-factor structure (12). This discrepancy may be attributable to sample characteristics (university students vs. broader population), cultural shifts in internet use, or methodological differences (our use of polychoric correlations and WLSMV estimation). Overall, the current results indicate that the one-factor model provided the most parsimonious admissible solution for the short forms, with both versions showing evidence consistent with unidimensional use in this sample.

Additional preliminary construct-validity evidence came from the associations between the short forms and the selected well-being indicators. The PIUQ-SF-6 and PIUQ-SF-9 showed very strong associations with the full PIUQ-18, confirming strong convergence with the full PIUQ-18. When examining non-overlapping correlations (correlating each short form with the remaining PIUQ-18 items not included in that short form), the PIUQ-SF-9 showed *r* = 0.70 and the PIUQ-SF-6 showed *r* = 0.60, providing more conservative estimates of shared variance. Both short forms exhibited theoretically consistent and highly similar associations with external variables to those of the full PIUQ-18. The short forms also demonstrated significant negative associations with multiple well-being indicators, closely mirroring the full scale. These findings provide preliminary evidence that the short forms retain associations with the selected well-being indicators while requiring fewer items than the original 18-item version. However, when evaluated against the standards proposed by Smith et al. (40) for short-form development, the present study provides only partial evidence. The PIUQ-SF-6 and PIUQ-SF-9 were evaluated with confirmatory factor analysis in an independent subsample, and both showed acceptable unidimensional fit and convergence with the full scale. However, three of Smith et al. (40) recommendations were not met: (a) the parent and short forms were not administered separately, so the convergence estimates are likely biased; (b) content-coverage analyses were not conducted; and (c) classification accuracy (cutoff scores, sensitivity, specificity) was not evaluated. And since the external criteria were also self-reported well-being measures rather than clinical or behavioral indicators of PIU-related impairment, these findings support further evaluation of the PIUQ-SF-6 as a brief research measure of general PIU symptoms, but do not establish its utility as a screening instrument.

## 5 Limitations and implications

Several limitations should be acknowledged. The generalizability of these findings is constrained by the sample characteristics. Data were collected from a single university in Hong Kong, with participants predominantly female (85.5%) and aged 18–25, which limits extension to adolescents, non-student emerging adults, older adults, or clinical populations. Furthermore, the data were collected in April–May 2019, prior to the COVID-19 pandemic, a period that substantially altered global internet usage patterns. Consequently, these findings may not generalize to post-pandemic cohorts, other geographic regions, or culturally diverse populations where the manifestation of PIU symptoms may differ. Future cross-validation in more representative and gender-balanced samples is essential before the alternative factor structure or the PIUQ-SF-6 can be recommended for use across populations or evaluated as a screening measure.

The bifactor models for full scale and short forms failed to converge or produced improper solutions, limiting our ability to assess the relative contribution of the general factor versus specific factors. Future research with larger samples may be able to estimate these models more reliably. Meanwhile, the Control Disorder subscale of the newly identified three-factor model demonstrated somewhat lower internal consistency (α = 0.70) and weaker associations with well-being measures compared to the Preoccupation and Neglect subscales. This may indicate that the Control Disorder items (Items 1, 4, 12, 14, 16) require further refinement for Chinese populations, or that the construct of ‘Control Disorder’ manifests differently across cultures. Future research should examine whether alternative items or revised wording would improve the measurement of this dimension. Furthermore, although a Simplified Chinese PIUQ-SF-9 exists (12), we retranslated the full PIUQ-18 for consistency, which limits direct comparability with the previous Chinese validation. Because both one-factor and alternative three-factor models were admissible, the preferred scoring structure remains uncertain and should be evaluated in independent samples. In addition, measurement invariance across gender could not be reliably tested in the present study because the confirmatory subsample was highly imbalanced. Since the PIUQ-SF-6 may be considered as a brief screening measure that would be applied across genders, this is a substantive limitation. We therefore make no claims about configural, metric, or scalar invariance, and we caution against using the present factor structure or short-form scores for between-gender comparisons until invariance has been established in a gender-balanced sample.

## 6 Conclusion

In this sample of Chinese university students, the original PIUQ three-factor model was inadmissible. An exploratory alternative three-factor model was the best-fitting admissible PIUQ-18 model, but its incremental fit was below conventional benchmarks, and its structure requires independent replication. The PIUQ-SF-6 showed the strongest evidence for a unidimensional short-form model, whereas the PIUQ-SF-9 showed weaker fit. The Control Disorder dimension also demonstrated weaker reliability and external validity evidence. These findings provide preliminary psychometric evidence but do not establish clinical validity or screening utility. Further research should examine the proposed structures in independent, more diverse, and gender-balanced samples and validate the measures against clinically or behaviorally meaningful criteria.

## Data Availability

The raw data supporting the conclusions of this article will be made available by the authors, without undue reservation.
